# Next-Generation Payment System for Device-to-Device Content and Processing Sharing

**DOI:** 10.3390/s22072451

**Published:** 2022-03-23

**Authors:** Fatih Kihtir, Mehmet Akif Yazici, Kasim Oztoprak, Ferda Nur Alpaslan

**Affiliations:** 1 Department of Computer Engineering, Middle East Technical University, Ankara 06800, Turkey; fkihtir@apprecode.com.tr (F.K.); alpaslan@ceng.metu.edu.tr (F.N.A.); 2 Informatics Institute, Istanbul Technical University, Istanbul 34469, Turkey; yazicima@itu.edu.tr; 3 Department of Computer Engineering, Konya Food and Agriculture University, Konya 42080, Turkey

**Keywords:** device-to-device communication, content delivery networks, mobile edge computing, incentive based resource sharing, peer to peer resource sharing, wireless communication, mobile devices

## Abstract

Recent developments in telecommunication world have allowed customers to share the storage and processing capabilities of their devices by providing services through fast and reliable connections. This evolution, however, requires building an incentive system to encourage information exchange in future telecommunication networks. In this study, we propose a mechanism to share bandwidth and processing resources among subscribers using smart contracts and a blockchain-based incentive mechanism, which is used to encourage subscribers to share their resources. We demonstrate the applicability of the proposed method through two use cases: (i) exchanging multimedia data and (ii) CPU sharing. We propose a universal user-to-user and user-to-operator payment system, named TelCash, which provides a solution for current roaming problems and establishes trust in X2X communications. TelCash has a great potential in solving the charges of roaming and reputation management (reliance) problems in telecommunications sector. We also show, by using a simulation study, that encouraging D2D communication leads to a significant increase in content quality, and there is a threshold after which downloading from base station is dramatically cut down and can be kept as low as 10%.

## 1. Introduction

Recent decades have witnessed a huge and sharp increase in the transmission of multimedia data. Although this is a direct consequence of the advances in communication technologies, the increase in the need for larger bandwidth, higher data rates, and larger processing power have surpassed improvements in the underlying technologies. The rapid increase in the need for higher data rates and better processing power has led researchers to consider unused resources at the edge of the networks to improve the performance of end user devices. A device can speed up the execution of its tasks by using the idle processing power of the devices in its proximity. Internet Service Providers can utilize the device-to-device (D2D) data transmission concept [1] to reduce data traffic load on its servers, backbone network, and access points, as well as to reduce the latency of accessing resources, which is meaningful especially for services with low latency demands. D2D data delivery can be used to deliver data from an access point to a single device and relay it to other requesting devices in the same network. Making use of these idle resources, however, comes with its problems [2], namely the following: coordination and housekeeping, privacy concerns, and unwillingness of the nodes to share their resources. In this study, we propose a D2D data delivery framework that employs a cryptocurrency-based payment system to provide incentives for sharing unused bandwidth and computation resources between devices.

Data streaming, specifically bulky multimedia data such as high definition videos requires vast bandwidth. D2D data transmission facilitates these types of applications. The requesting device sends its request to the access point, which maintains a list of current users of the stream. If a device downloading the stream is interested in providing it to the devices in its signal range, the access point informs both devices to establish the connection.

Another important parameter regarding multimedia communications is latency, which can also be considered one of the major reasons hindering the usage of Internet of Things (IoT) in time-restricted applications or applications running on networks with limited bandwidth connections where the latency mainly stems from sending data to cloud servers and processing them there. Some applications include data coming from thousands of sensors, which report the current status of the systems under control for which transmission to the cloud is not necessary. As a solution to this problem, processing at local servers has been proposed. This solution entails the usage of micro-processing power available at end-user devices in a distributed form. It encourages end users to share the processing power of their devices in a local network, where the initial processing of data can be carried out, and the need for forwarding the data to cloud will mostly be eliminated. Imagine that you have tens of sensors collecting data from a building’s security system. The use of local computing power of a node within the cell would help you to process raw data with the ability to access them at high speeds. Then, the peer would send a report (processed result) to the owner of the data, which then could be sent to the network operation center. Such scenarios are the promise of Edge Computing. Edge Computing [3] involves assigning a task to a device, processing the task, and sending back the results to the requester. In a similar manner to data sharing, edge processing requires scheduling, solving security and privacy problems, and, more importantly, providing incentives for the owners of the devices to share their computing resources. Edge computing incentive system is one of the use-cases that we consider for our proposed system. P2P video streaming systems suffered the mechanisms of managing incentives before the rise of block-chain based systems [4,5].

In this study, a framework is proposed to share the unused bandwidth between the devices, as well as the computing power, in a network for reducing the total traffic load of the servers and access points. We investigate the cost of receiving data from other users, as opposed to receiving it from the access point. This can alternatively be interpreted as the revenue of providing data to the other users. We look at several scenarios that impact the behavior of the users, the amount of currency exchanged, and the quality of the content users can access. The payments, which are the incentives provided to the users for sharing their idle resources, are executed as cryptocurrency.

In this study, we propose a mechanism to share bandwidth and processing resources among subscribers by using smart contracts and a blockchain-based incentive mechanism, which is used to encourage subscribers to share their resources. The proposed incentive system will be a means to encourage and enhance information exchange among multiple parties in future telecommunication networks.

We demonstrate the applicability of the proposed method by examining several use cases:

1 Exchanging multimedia data by simulating video streaming playout scenarios around a macrocell;
2 CPU sharing for video transcoding that the requesting parties need;
3 Increasing the quality of playout in the network.

We propose a framework for the use of a universal payment system named TelCash [6] between the users, and it will potentially be used later between the users and operators, which propose a solution for current roaming problem and establishes trust in X2X communications. TelCash has great potential in solving the charges of the roaming problem in the telecommunication world by allowing subscribers to pay the fees for the services purchased from any operator using crypto-cash. The progress in the usage of e-sim technology is one of the motivators of this idea. However, it should also be noted that the common platform where all operators and users converge plays an important role in the authentication of an unknown subscriber to a new operator.

The contributions of this study can be summarized as follows:

- A framework inspired from a recently described crypto-currency-based payment system, TelCash, is proposed.
- The performance of a single-cell system using this framework with the goal of maximizing content quality is investigated. Quantitative results are obtained, which demonstrate that the quality of the content significantly increased and also maintained even when trans-coding is not used.
- The effect of the cost attached to the content obtained from the base station is quantified, which can pave the way toward analyses in pricing strategies from the perspective of mobile operators and content providers.

The rest of this paper is organized as follows: In the next section, we present a literature survey to inform the readers about the state-of-the-art studies in such systems. Next, in Section 3, we describe the system model our analysis is based on. We present the results from our simulation study in Section 4. Finally, we conclude with Section 5.

## 2. Background

Cryptocurrency and distributed ledger technologies have been attracting the attention of both research and business communities since their inception. The advent of distributed ledgers enabled enterprises and supply chains with the opportunity to delegate trust to a third party, which is a single source of truth, in a decentralised manner. As an abstraction, we may consider a ledger as a collection of data items that are replicated and distributed over different locations. These data structures do not require any central administration. Instead, consensus systems, such as proof of work, proof of stake, or virtual voting, are employed to ensure that all replicas are exactly alike. A peer-to-peer network is used as the underlying infrastructure for accessing data. The internal organization of the data may not be necessarily the same in all implementations. The enabler for distributed ledgers is the blockchain concept [7,8] and there are many specifications for blockchain such as Hyperledger [9,10], an open source project supported by Linux foundation, and the Hedera project [11], which places more emphasis on the efficiency of processing data. These mainly differ in their consensus algorithms, security mechanisms, and transaction speed. The Hedera project is especially promising in that it offers several orders of magnitude more transactions per second compared to other blockchain systems due to the difference in the gossip algorithm and its unique chainless distributed ledger called hashgraph, which makes Hedera a good candidate for micro-transactions that are needed between the users in the proposed framework.

The idea for cryptographic electronic money was conceived in the early 1980s [12,13], and early implementations appeared in the 1990s. Bitcoin [14], the first decentralized cryptocurrency, went online in 2009 and has made a huge impact since then. The list of use cases and potential applications for cryptocurrencies is ever-growing, and it has gained acceleration due to the imminent migration to 5G cellular communications. In this context, cryptocurrency can be seen as an enabler for smart home and smart city applications and Internet of Things (IoT)-based systems as well as enabling a payment scheme for 5G infrastructure coverage and use. Coupled with cryptocurrency, distributed ledger technologies has become powerful tools and platforms. Among its potential applications, one can list the following:

- Smart contracts, where financial statements can be exchanged securely and truthfully, and the obligations of the contracting parties are verifed automatically [15,16];
- Smart instruments that connect to the Internet and provide you more information about their applications and the surroundings [17]. These kinds of applications can also be extended to build communication infrastructure for different technologies (IoT, 5G, etc.);
- Supply chain transformation with blockchain integration [18];
- Healthcare networking, where only authorized entities are provided access to personal records that are secured with blockchain [19];
- Artistic and intellectual property rights as well as digital rights management through blockchain [17];
- E-voting [20];
- Identity management by allowing only the necessary parts of personal information to be viewed by the requesting people, encryption of one’s credentials, and record keeping for documents such as birth, marriage, and death certificates [21].

Cryptocurrencies and distributed ledger technologies have observed an eruption in popularity since the release of Bitcoin whitepaper [14]. Despite being the most prominent cryptocurrency, Bitcoin has a number of issues. Its mining process involves presenting a proof-of-work (PoW) in a new block, which includes finding a nonce value for the last block of the public ledger. This is a very energy-consuming process, requiring 204.5 KWh per transaction on average, which amounts to 204.5 TWh yearly consumption [22]. Furthermore, the confirmation of a transaction requires a relatively long latency (ranging from about 20 to 200 min on average, occasionally exceeding 17 h around February 2018) [23]. This duration is relatively small and is around 13.72 min on average as of February 2022 [24]. The limit on the size of the blocks is another issue that increases transaction fees and transaction processing time. It is estimated that a switch to proof-of-stake could save 99.95% of the energy currently required to run a proof-of-work based system [23] in addition to the reduction in processing time.

In light of these observations, many alternative digital currencies, namely “altcoins”, have been designed and have started circulation. Ethereum [25] is an open-source distributed platform based on blockchain and a virtual machine that runs on the distributed ledger. The advantages of Ethereum over the technology behind Bitcoin include improved block processing time (around 15 s, as opposed to Bitcoin’s 10 min on the average [26]), unlimited block size, and the perpetual addition of “Ether” to the system in contrast to the ultimate limit on the total amount of Bitcoins mineable and the “smart contracts”, which interact with the ledger using a domain-specific language. LiteCoin [27] is a form of BitCoin and claims that the transaction confirmation time is almost zero, making it a strong candidate for real-time payment applications [28]. Nano [29], another altcoin, differs from the others in that it uses Proof of Stake (PoS) instead of PoW in order to avoid power-consuming computations. Another cryptocurrency designed for facilitating financial operations without any banking system involved is TelCoin [30], which considers the subscribers of mobile operators as the potential users. A subscriber of a member mobile operator is able to transfer or receive TelCoin to or from other subscribers. TelCoins can be obtained by transferring from other members, or they can be bought directly from the mobile operator, unlike other cryptocurrencies where mining is normally the only method to produce currency.

The security aspect of blockchain contracts in IoTs are currently in the focus of the researchers. In one of the recent papers [31], the authors focus on the security aspects of smart contracts in IoT use-case scenarios and found out that their model established a firm architecture to be used in real life. In this work, we concentrate on blockchain-based incentive mechanisms to be used in real-life scenarios, without disregarding the security, reliability, and usability properties of a typical blockchain.

Scarcity of resources presented people with an opportunity to convert their idle resources into economic value. This idea led to house sharing, car sharing, bicycle sharing, and even wireless network sharing [32]. Mobile device bandwidths are limited resources, but at the same time, they may sit idle for a long time before being utilized.

As mentioned earlier, in one of the early papers [32] on blockchain and cryptocurrencies, researchers investigated the possibility of resource sharing in a wireless mesh network. They stated that, “The idea of the compensation system is to create a balance between total resource contribution and its consumption. The economic value of the contribution and consumption of network resources for each participant in a given locality are recorded”. In their reference architecture, bandwidth-sharing was performed manually, and they made it fully automatic, rule-based sharing using a controlled blockchain deployment.

Use of blockchain in telecommunication networks is particularly attracting attention in recent years. We have compiled a list of articles and compared the related work in Table 1. These have been selected from the search results on Google Scholar from 2018 onward using the following search phrases: “blockchain and telecommunications”, “blockchain and incentive”, “blockchain and iot”, and “blockchain and device-to-device”.

From Table 1, it can be concluded that our work combines the five important features relevant to the use of blockchain in telecommunication networks. CC column identifies whether the related work includes a cryptocurrency creation. We have come across works that involve new cryptocurrency generation, although one work does not create a new cryptocurrency but utilizes an existing one. The IB column identifies whether the related work includes an incentive-based approach. This type of separation is needed because cryptocurrency generation does not always automatically include an incentive-based approach. The Telco column identifies the research that has applications in telecommunications. Except [33], all works are directly or indirectly related to telecommunications operating domain. By resource-sharing, we refer to the works involving a framework that rewards the end-user for sharing their resources. Finally, the last column labeled D2D points out whether the research includes device-to-device communication or not. The most relevant work to our setting seems to be [40], which includes incentive-based, accountable infrastructure sharing in 6G networks, but it does not propose any kind of cryptocurrency generation, rather moving forward with transparent accountable metering.

Although the first study proposing D2D communication appeared in the literature in 2000 by [41], in order to enable multi-hop relays in cellular networks, the area gained interest after 2010. The main idea in D2D communication is to increase the efficiency of the limited resources in the cellular spectrum. Although there exist sparse use cases of D2D, the research in the area can be categorized into two main categories: (i) in-band and (ii) out-band communication [1]. In the former, both D2D and cellular communication takes place in the same channel. The D2D communication uses unlicensed spectrums and different communication architectures such as LTE-A, Wi-Fi, and Bluetooth in the latter. While the researchers working on the former mostly study interference management and resource allocation, the researchers working on out-band communications concentrate on solving management problems of multiple channels and multiple radio interfaces.

Successfully architectured D2D communication increases the performance of cellular networks in multiple ways. While the throughput of the system increases up to 300% in in-band communication [42], it increases up to 100 times in out-band communication [43]. Ref. [44] proposed a hierarchical routing algorithm to increase the lifetime of the sensors, alternatively reducing unit energy consumption.

Recently, massive data transfer and energy efficiency is gaining the attention of researchers. Ref. [45] proposed a content distribution architecture utilizing out-band channels in addition to a cellular network. The study shows that the proposed architecture reduces energy consumption by a factor of 8, while increasing the throughput 50-fold by utilizing a caching mechanism on the base station and sharing video content with nearby devices/peers. Similarly, ref. [46] proposes a three-layer hierarchical content access mechanism allowing users to access to the local cache, specifically constructed cache on the storage area of other devices, or from the serving base station through the backhaul transmission. Since they designed a distributed caching mechanism, they preferred to use truncated Zipf distribution on cache replacement rather than optimizing the cache placement algorithm. They used a Genetic algorithm with two-step search in order to achieve the expected outcome.

Ref. [47] proposes to build an overlay network by dynamically detecting the physical network infrastructure and manage communication resources by the information obtained from D2D communication. The study demonstrates the success of content awareness among the users and 27.5% better task offloading time when compared with random placement. The authors also propose using either game-based, pricing-based, or contract-based incentive mechanisms via their Knowledge Centric Edge (KCE) computing services.

One of the biggest challenges in D2D communication is the willingness of the operators to have control on the communication. A successful platform allowing users to share data among themselves should find a way to allow operators to be involved in some manner. The focus of this study is to propose an incentive mechanism to bring users and operators to a common platform as a stakeholder.

## 3. System Model

We consider a single cell with *N* mobile users. A number of the users are assumed to form social clusters around attraction points, such as popular shops in a mall or classrooms in a university campus. The attraction points are assumed to be located randomly with a uniform distribution within the cell, which has a radius of *R*. Similarly, mobile users within a social cluster are located randomly with a uniform distribution within the cluster radius, r<R. There are also free-roaming mobile users that do not belong to a social cluster. In this study, we assume that users are stationary and leave the analysis of the impact of mobility to future studies.

We assume that each video has a number of different quality copies at the content server. A mobile user requesting the video will be able to play the video in a quality level determined by its downlink data rate. The downlink data rate that a mobile user achieves depends on the distance between the mobile user and the source. In this study, we assume a piece-wise constant dependence in the following manner: If the distance between the mobile user and the source is below a certain threshold, $d_{1}$, the best-quality video is downloaded. If the distance is between $d_{1}$ and a second-tier threshold, $d_{2}$, the second-best-quality video is downloaded and so forth up to the cell diameter, 2R. A conceivable scenario in this context is that communication around the attraction points is over WiFi, whereas either licensed or unlicensed cellular channels are employed for transmissions over longer distances.

Each video is assumed to be encoded in group-of-pictures (GOP) structure, where we assume constant GOP length. In this manner, we can define the video length in terms of the number of GOPs. There are three types of videos:

1 Short videos: These have lengths uniformly distributed between $L_{s,min}$ and $L_{s,max}$ and are played out entirely in sequence.
2 Long videos: These have lengths uniformly distributed between $L_{l,min}$ and $L_{l,max}$, where $L_{s,max}<L_{l,min}$, and they are played out entirely in sequence.
3 Seek videos: These have lengths uniformly distributed between $L_{l,min}$ and $L_{l,max}$ similarly to the long videos. However, we assume that the viewer skips through certain portions of this type of videos. After each GOP is viewed, the next GOP is played out with probability $1-p_{seek}$, whereas the viewer seeks uniformly one of the GOPs in the remainder of the video after this particular GOP with probability $p_{seek}$.

Each video is requested by each mobile user at most once. Associated with each video file *i* is a “popularity index”, $p_{i}$. The arrival of the requests to video file *i* follows a Poisson process with intensity $\lambda_{i}$ that is proportional to $p_{i}$.

Upon the request of a video file, the mobile user informs the base station. If the requested content is present neither in the cache of the base station nor in any of the mobile users, it is downloaded from the cloud/content server and cached at the base station. In the mean time, the related virtual network function (VNF) co-located at the same computing unit hosting the base station keeps track of the cache of each mobile user and maintains a map of each GOP of each video file. Based on this, the base station informs the requesting mobile user of the other mobile users that have a copy of the next GOP to be played out, as well as their geographical locations. In the light of this information, the requesting mobile user decides the location to download from.

If one of the mobile users is selected as the source for the next GOP to be played out, there is a cost attached to it. This cost roughly reflects the energy to be consumed by the provider user for this process. The energy required for transmission of a signal over a distance of *d* is inversely proportional to $d^{k}$, where *k* ranges from 2 to 4, depending on the scenario [48]. In [49], the energy required to transmit an *L*-bit packet over a distance of *d* is given by the following:

(1) $$E\left(d,L\right)=E_{te}\;L+E_{ta}\;L\;d^{2},$$

where $E_{te}$ is the energy spent in the transmitter electronics per bit, whereas $E_{ta}$ is the energy spent in the transmit amplifier per bit per squared distance. Typical values for $E_{te}$ and $E_{ta}$ are given in [49] as 50 nJ/bit and 100 pJ/bit/m${}^{2}$, respectively. This shows that for transmission over short distances, the energy spent in the transmitter electronics is dominant, whereas the energy spent in the transmit amplifier becomes the significant component as distance grows. Based on this observation, we model the cost of data provision over a distance of *d* as follows:

(2) $$C_{1}=\max\left\{c_{p}\;d^{k},c_{\min}\right\},$$

where $c_{p}$ is the coefficient that determines the cut-off for *d* where the energy spent in the transmit amplifier becomes dominant, and $c_{\min}$ represents the minimum cost representing the case where the energy spent in the transmitter electronics is dominant.

Furthermore, consider the scenario where the mobile user selected as the source has the requested GOP only in the best-quality available, whereas the distance between the requesting user and the source user does not allow best-quality data rate but the second-best-quality. In this case, the source user transcodes GOP into the second-best quality and transmits the output. There is a further cost associated with this video processing that the requesting user has to pay in addition to the cost of merely providing the content. We model the transcoding cost from the quality level of the source, $q_{s}$, to that of the destination, $q_{d}$ (where smaller value indicates better quality), as follows:

(3) $$C_{2}=c_{t}\;\left(q_{d}-q_{s}\right),$$

where $c_{t}$ represents a factor relating data provision to transcoding cost. In this case, the total cost the requester needs to pay the provider is as follows.

(4) $$C=C_{1}+C_{2}.$$

Note that, in this scenario, the second-best quality GOP will now be present and available to be downloaded from the source user’s cache should there be requests for it later. In this manner, it is possible that mobile users host multiple copies of the same GOP in different quality levels. The cost of data provision as well as the cost of video processing, when applicable, is paid to the source user by the requester via TelCash.

The requesting mobile user picks the source based on the quality first, then cost, and then the distance. As soon as it learns the source candidates from the base station, the requester computes the quality level that it can obtain from each source as well as the base station, based on the cache maps of each device along with the distance information. Then, among the mobile users that can provide the highest possible quality, the one that demands the least cost is determined. If there are multiple such users (including the base station cache), the closest one is preferred. Finally, the quality level that can be offered by the base station and the associated cost is compared against the source candidate mobile user. We model the cost of downloading from the base station (either from the cloud or the cache at the base station) as follows:

(5) $$C_{\mathrm{BS}}=c_{b}\;\max\left\{c_{p}\;d^{k},c_{\min}\right\},$$

where *d* here is the distance between the requester and the base station. Equation (5) differs from (2) only in the coefficient $c_{b}$, which is a parameter related to the willingness of the mobile operator providing content that has already been brought down from the cloud. Note that although $c_{b}=0$ represents the current commercial practice, this could change soon with the deployment of 5G and the ever-growing trend in the content delivery use-cases. After this comparison, if the base station beats the best candidate user in terms of quality, cost, or distance in that order, GOP is downloaded from the base station.

Upon reviewing the literature on different pillars such as using Cryptocurrency, Incentive-Based mechanisms, Resource-Sharing, ability to work on D2D systems, utilizing maximum channels such as bundling licensed and unlicensed channels, and energy usage optimization, the proposed scheme seems to be complete in utilizing all necessary components for a holistic approach.

The experimental studies or implementations in the literature encourages us that the proposed model in this section can work well in production, in addition to the simulation results presented in next section. The performance improvements in distributed public ledger technologies is expected to pave the way for the success of the proposed scheme, which can also find significant uses in the economy produced by newly emerging gaming applications based on fungible tokens.

## 4. Simulation Study and Numerical Results

A standalone simulator has been written in C++ for this study. Each video is requested by each mobile user at most once during the simulation. The arrival of the requests to video file *i* follows a Poisson process with intensity $N\;p_{i}/T$, where $p_{i}$ is the “popularity” of video file *i*, and *T* is the duration of simulation. The popularity $p_{i}$ is, in fact, a probability that has Zipf distribution [50]. The exponent of the Zipf distribution is selected so that the resulting distribution yields the well-known Pareto (90/10) law [51,52] among the video population. In this manner, 90% of the requests are for the most popular 10% of the contents on the average. In this study, we define 10 popularity classes, where each class has an equal number of mobile users belonging to them. The popularity of each class is determined according to the described Zipf distribution. The mobile users that request each video are selected randomly according to a uniform distribution. This means that on average, $N\;p_{i}$ mobile users will request video file *i*, where the popularity class video file *i* belongs determines $p_{i}$, and the arrival of the requests obeys a Poisson process.

The simulation parameters are summarized in Table 2. The number of the quality levels of the videos is set to be three. The duration of the video files was taken upon the considerations of Internet dynamics. A typical maximum twitter video length is 140 s; hence, the minimum video size is set to five GOPs (or 10 s) in the short video group while the maximum size is set to be 70 GOPs (or 140 s). Similarly, minimum and maximum values for long videos are set to be 150 GOPs (5 min) and 450 GOPs (15 min), respectively.The quality level a user receives a video depends on the distance of the user to the source it is receiving the content from, whether it be the base station or another user. The threshold for the highest quality level is set to $d_{1}=90$ m, i.e. the user will receive the highest quality available if its distance to the source is less than 90 m, whereas the threshold for the second quality level is set to $d_{2}=300$ m.

**Figure 1:**
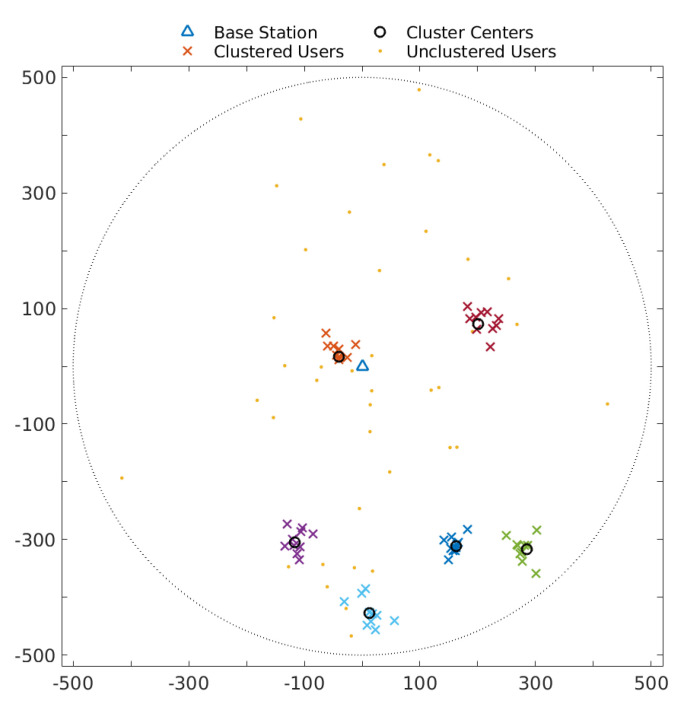
Sample topology. Axes represent distance in meters.

A sample topology the simulation is run on is provided in Figure 1. There are six user clusters, representing attraction points where users tend to gather together, each consisting of 10 users, centered around the cluster centers indicated by the small circles and uniformly distributed within the cluster radius, r=50 m. In addition, there are 40 more users that do not belong to any cluster. These are uniformly distributed within the entire cell, which is assumed to be a circular region of radius R=500 m.

**Figure 2:**
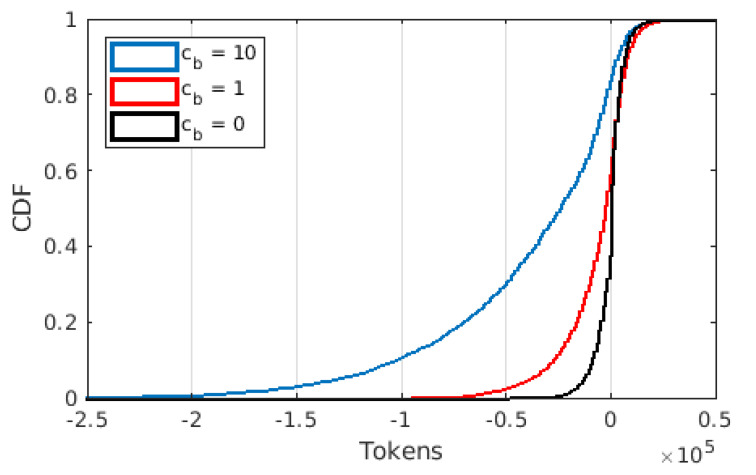
Cumulative Distribution Function of token balance for different values of $c_{b}$.

The distributions of the token balance are plotted in Figure 2 for $c_{b}=0$, 1, and 10. Observe that $c_{b}=0$ corresponds to current commercial practice in which no extra cost is attached to downloading from a cloud/base station. (In fact, due to the data quotas users have on their plans and charges on excess data, it is not entirely accurate to say that $c_{b}=0$ for current practice. One could argue that $c_{b}$ is effectively >0 under current practice, but within the quota, it could be said that downloading content from the base station is free). In this setting, we see a lot of users with small balances, which illustrates heavy downloading from base station, which makes sense as the cost in this case is 0. As $c_{b}$ increases, users are discouraged to download from the cloud/base station whenever possible. With growing $c_{b}$, the proportion of users with balance close to 0 decreases. This is expected, as more users try to download from other users. Moreover, in this scenario, we see many users paying excessive amounts of tokens for content, whereas we do not see as many users that capture the demand and make excessive amounts of gains. In this study, we ignored the scenarios where a user cannot download content from other users and/or the cloud/base station due to a lack of credit, hence permitting negative balances. This illustrates the expenditure of users under the given scenarios. Further studies need to be conducted to determine optimum pricing policies for the providers, which is left out of the scope for this study.

**Figure 3:**
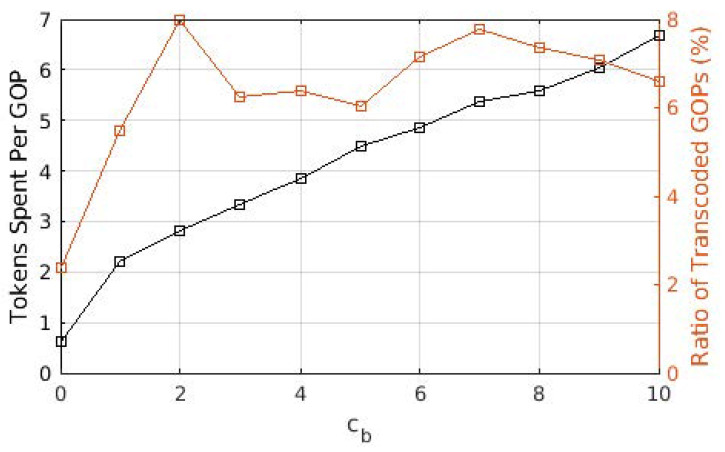
Tokens paid per GOP for different values of $c_{b}$.

**Figure 4:**
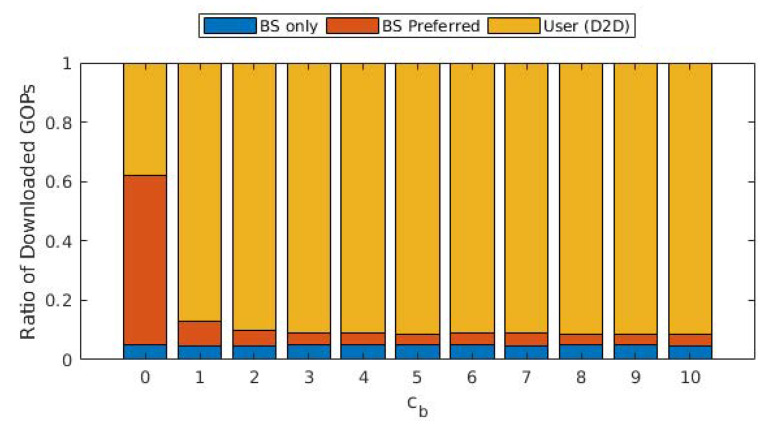
Ratio of the GOPs downloaded from the cloud/base station (blue: GOPs that do not exist in the cache of any of the users and downloaded from the cloud; red: GOPs downloaded from the base station cache), and GOPs downloaded from other users for different values of $c_{b}$.

We present average tokens paid for each GOP in Figure 3, as well as the ratio of GOPs that required transcoding from the source user. The average cost of a GOP increases threefold when $c_{b}$ increased from 0 to 1, whereas it increased almost linearly with a lower slope when $c_{b}>1$. A similar effect can be observed with the ratio of GOPs that required transcoding. When $c_{b}=0$, a significant portion of GOPs is downloaded from the cloud/base station, which requires no transcoding. On the other hand, as the cost of downloading from the cloud/base station increased, the share of GOPs downloaded from other users increases, hence increasing the ratio of GOPs that required transcoding. For $c_{b}>1$, this ratio varies between 6 and 8%, as opposed to the 2% for $c_{b}=0$. This is in accordance with the ratio of the GOPs downloaded from the cloud/base station, which is provided in Figure 4. The portion labeled “BS only” is due to the GOPs that are being downloaded for the first time in the entire cell and, thus, does not exist in any of the users; therefore, they are bound to be downloaded from the base station. This ratio does not change with $c_{b}$, which is to be expected as the arrival process and the distribution of the video files are not affected. This figure demonstrates that (i) even with the base station providing free content, almost 40% of the GOPs are downloaded in D2D mode since the objective is to maximize quality, and (ii) as soon as $c_{b}$ becomes ≥1, downloads from the base station are severely limited, and the exact value of $c_{b}$ has little effect on the ratio.

**Figure 5:**
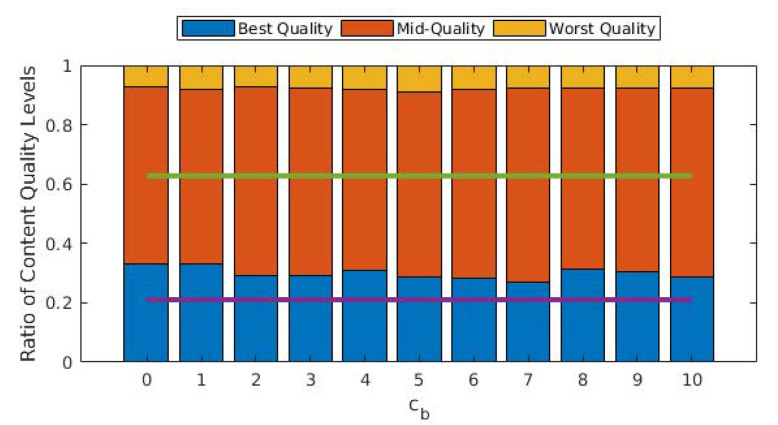
Ratios of the GOPs with respect to quality for different values of $c_{b}$. Purple and green lines show the best and (cumulative) mid-quality ratios when D2D mode is disallowed.

In Figure 5, we illustrate the ratio of the quality levels of the GOPs that are played out. One conclusion that can be inferred from this figure is that the average GOP quality is not significantly affected by $c_{b}$. More importantly, allowing D2D modes significantly improves the average GOP quality, as almost 40% of all GOPs are played out in the worst quality level when the D2D mode is disallowed. The improvement in the share of best-quality GOPs might seem limited, but the share of worst-quality GOPs is drastically decreased with D2D.

**Figure 6:**
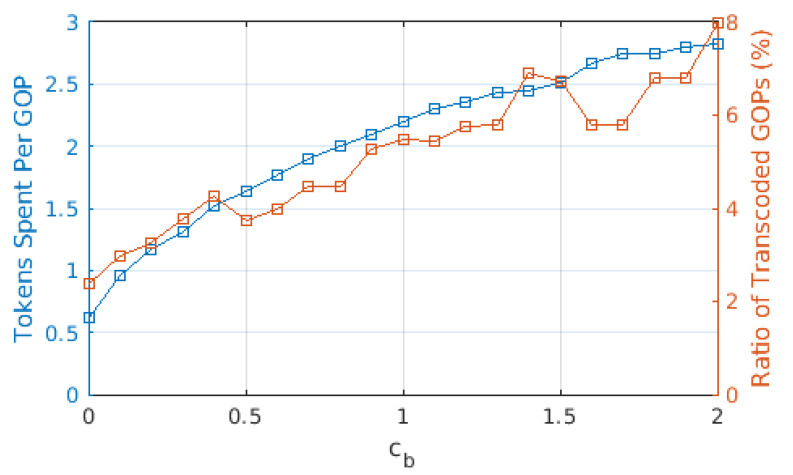
Tokens paid per GOP for different values of $c_{b}$.

**Figure 7:**
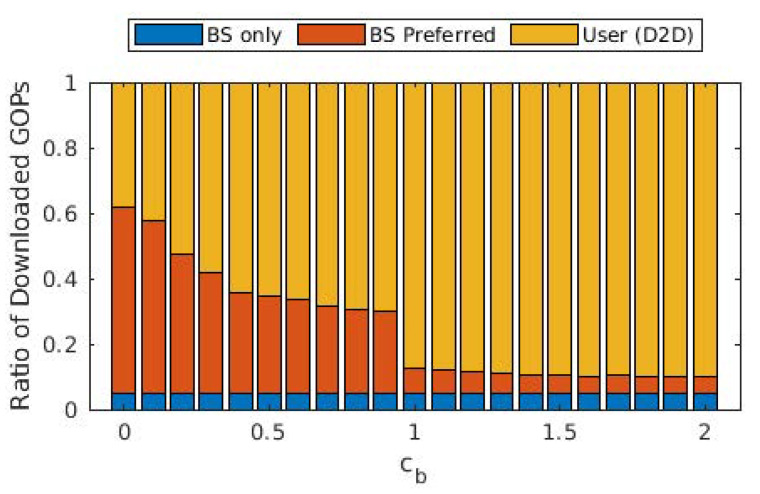
Ratio of the GOPs downloaded from the cloud/base station and GOPs downloaded from other users for different values of $c_{b}$.

**Figure 8:**
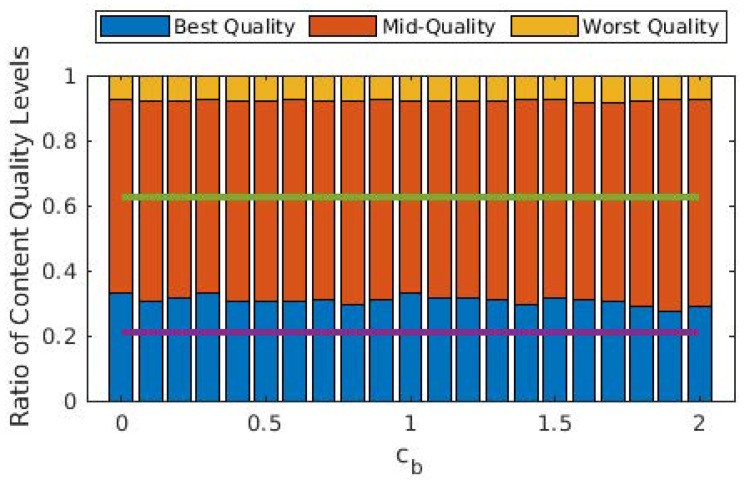
Ratios of the GOPs with respect to quality for different values of $c_{b}$. Purple and green lines show the best and (cumulative) mid-quality ratios when D2D mode is disallowed.

From Figure 3, Figure 4 and Figure 5, we observe that the behavior of the system does not drastically change when $c_{b}>1$. In order to observe performance in a more granular fashion, we plot the same graphs for $0\leq c_{b}\leq 2$ with steps of 0.1 in Figure 6, Figure 7 and Figure 8. The cut-off at $c_{b}=1$ can be observed much better in Figure 7 and Figure 8. As $c_{b}$ approaches 1, the ratio of the GOPs downloaded from the base station gradually decreases. However, we observe a sudden drop at $c_{b}=1$, at which point the base station becomes just another user from the perspective of the mobile users. After this point on, the performance of the system in terms of the number of GOPs downloaded from the base station and quality distribution does not change significantly. In fact, across all $c_{b}$ values, the quality distribution of the GOPs more or less stays the same as the objective is to maximize quality; hence, the same quality level is maintained throughout, possibly with an increase in cost per GOP.

Finally, we experimented with the same set of parameters presented in Table 2 by disallowing transcoding this time. Our results show that token balance distribution does not deviate significantly from the case where transcoding is allowed (and thus, the figures were omitted).

**Figure 9:**
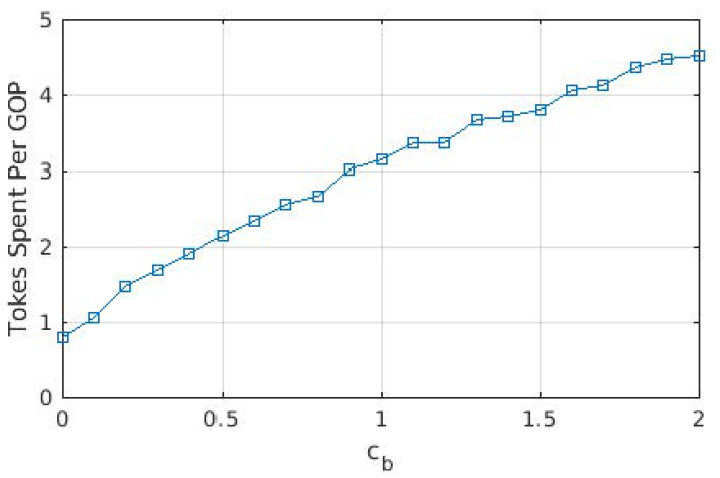
Tokens paid per GOP for different values of $c_{b}$ when transcoding is disallowed.

**Figure 10:**
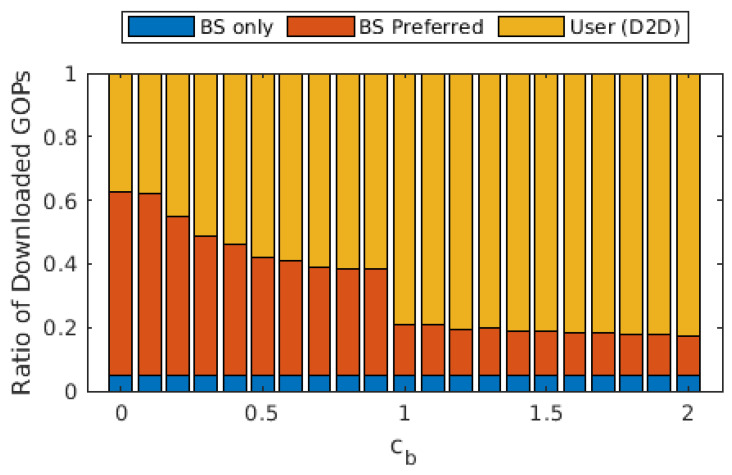
Ratio of the GOPs downloaded from the cloud/base station and GOPs downloaded from other users for different values of $c_{b}$ when transcoding is disallowed.

**Figure 11:**
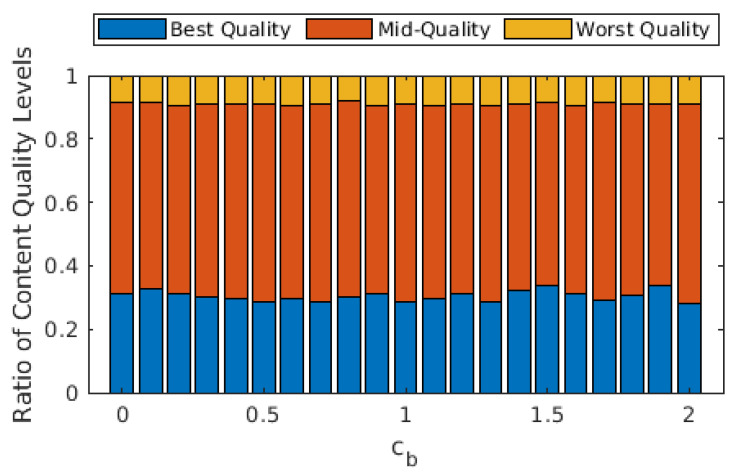
Ratios of the GOPs with respect to quality for different values of $c_{b}$ when transcoding is disallowed. Purple and green lines show the best and (cumulative) mid-quality ratios when D2D mode is disallowed.

When compared to Figure 6, Figure 9 reveals that cost per GOP is increased when transcoding is disallowed. Transcoding required means that the source will need to downgrade its content due to the distance between itself and the requesting user, meaning that the requesting user cannot have the best quality anyway. When transcoding is disallowed, to obtain the best possible quality, a user has to download from a user that is somewhat distant to it, which in turn adversely affects cost due to Equation (2). On the other hand, Figure 10 and Figure 11 show that the same quality level is maintained when compared to the case where transcoding is allowed. Consequently, one can argue that disallowing transcoding may lead to a loss in quality in a setting where users also take into account the cost of the transaction.

## 5. Conclusions

In this study, we propose a framework for enabling content and process power sharing between mobile users in a next-generation cellular network. This framework also has the potential to be used:

- Among users: to enable content and processing power sharing;
- Between users and the mobile operator: for purposes of charging based on specific content and/or application use;
- Between users and the content provider: for purposes of charging based on content access;
- In a system involving mobile users and multiple operators and/or providers, enabling data roaming and content sharing across domains;
- As a reference model for studies on reliability/availability of content in P2P-like systems where content is obtained through other users.

Based on a simulation study of a single cell system in which users prioritize content quality (in terms of video quality in the specific scenario investigated), we have observed the following:

- Content quality on the average is insensitive to the charge of downloading from the base station, and the average quality is greatly improved when the D2D mode is enabled;
- the charge of downloading from the base station affects the average cost of a unit piece of content, as there will always be a first time where content will be delivered from the content provider/cloud, which naturally is served through the base station;
- Even when the content from the base station is free of charge, a significant amount of content is downloaded from the users instead of the base station in order to achieve the best possible quality level;
- The ratio of the content downloaded from the base station can be kept to levels as low as 10% when the cost of downloading from the base station is no less than that of downloading from another user;
- Allowing transcoding has little effects on the average quality of the content, but it can decrease the average cost of the content.

The performance of the proposed scheme is investigated via a simulation of scenarios involving attraction points, such as shops in a mall, or restaurants, or social events. The concentration of users around these points is assumed to be higher, whereas free-roaming users are also allowed. In scenarios where users clustered together are interested in the same content such as videos from a sporting event or a disaster area, allowing users to exchange data directly alleviates a significant amount of load from the base station.

The second use case of the proposed scheme would arise when traveling abroad. Rather than paying a considerable amount of roaming fees, the users who are part of this system can gain access to content provided by peer users existing in their vicinity using their TelCash balance and could even provide access to their content if there is demand, earning TelCash. In the usual operation of roaming systems, the roamer’s data requests are directed to their origin country, and the response to their requests are routed through the same path, incurring long latency, which is very undesirable for multimedia content. The proposed method can improve latency performance in such scenarios.

Another aspect to be addressed for such systems is the integration to the tax system of the governments. Since crypto-currency payment systems are still in the gray-zone of tax systems and the governments are trying to install regulations for such systems, the solution/requirements for the governments would easily be integrated to the system.

Further studies can reach several directions. Discovery and advertisement for the services to be provided by the users could be defined more specifically. Devices within the D2D range should be discoverable and configurable easily. One aspect can be studying more complex and dynamic scenarios where users have mobility, multiple cells interact, and users may leave and enter cells. Another issue that needs further elaboration is the details of the enabling protocol running at the user’s station and the base station. At the base station side, this protocol will be responsible for keeping track of the cache map of the content at each user, whereas it will make decisions on what sources download which piece of content at the user’s side. The challenge with the design of this protocol would be keeping control messaging to a minimum. Moreover, the integration of WiFi into this framework calls for a more through investigation, particularly from the perspectives of energy consumption and channel efficiency and capacity. Finally, another important direction of research is pricing, which entails a study on determining the cost parameters related to downloading from users and the base station, the effect of the distance, and the cost attached to sharing processing power (which corresponds to transcoding in our proposed model). This will definitely shape the decisions of the users as some users inevitably would prioritize cost over quality. A study into the user behavior with budget constraints can reveal trends in such a system. The research on calculating the maximum capacity would also help decision makers to configure services dynamically, similarly to study [53]. Designing new service chain mechanisms in order to reduce the investment to core systems and integrating the proposed system with dynamic hardware run-time libraries to enable such collaborative systems would bring more intelligent solutions while increasing total performance [54].

## Figures and Tables

**Table 1:** Contemporary studies involving cryptocurrency-based system studies. CC: Crypto-Currency; IB: Incentive-based; Telco:Telecommunications Area; RS: Resource-sharing; D2D: Device-to-Device Communication.

Researchers	CC	IB	Telco	RS	D2D
Sankar et al. [33]	Yes	Yes	No	No	Yes
Khalid et al. [34]	No	Yes	Yes	No	Yes
Aryal [35]	No	No	Yes	No	No
Yousafzai et al. [36]	Yes (Existing)	Yes	Yes	Yes	No
Wang et al. [37]	No	Yes	Yes	No	No
Xenakis et al. [38]	No	Yes	Yes	No	No
Ribeiro et al. [39]	Yes	Yes	Yes	No	No
Faisal et al. [40]	No	Yes	Yes	Yes	Yes
Selimi et al. [32]	Yes	Yes	Yes	Yes	No
Our Work	Yes	Yes	Yes	Yes	Yes

**Table 2:** Simulation parameters.

Simulation duration	5000 s
Simulation runs per each data point	50
Number of video files	600
Number of Zipf popularity classes	10
Number of mobile users, *N*	100
Cell radius, *R*	500 m
Cluster radius, *r*	50 m
Number of clusters	6
Number of users per cluster	10
Number of video quality levels	3
Quality level distance thresholds	$d_{1}=90$ m, $d_{2}=300$ m
Short video length parameters	$L_{s,min}=5$ GOPs, $L_{s,max}=70$ GOPs
Long (and seek) video length parameters	$L_{l,min}=150$ GOPs, $L_{l,max}=450$ GOPs
Group of pictures (GOP) duration	2 s
Seek probability, $p_{seek}$	0.05
Energy loss exponent, *k*	2
Data provision cost coefficient, $c_{p}$	$10^{-4}$ token/m${}^{2}$
Minimum data provision cost, $c_{\min}$	1 token per GOP
Transcoding cost coefficient, $c_{t}$	1
Base station cost coefficient, $c_{b}$	varied between 0 and 10

## Data Availability

Not applicable.
